# Mr. Knowles in Reply to an Essex Practitioner

**Published:** 1812-05

**Authors:** E. L. Knowles

**Affiliations:** Soham


*To the Editors of the Medical and Physical Journal.*

GENTLEMEN,

IN looking over your Journal for this Month, I met with a paper, signed an Essex Practitioner; upon my reply to the Norfolk Practitioner, in your work for January, he says, my reply, in a great measure, consists of interrogatories, some of which he will not pretend to meddle with. E. P. had better not have made any observations, if he was not able to answer the whole decidedly and satisfactorily. He further remarks, " but, when he asks the N. P. whether he has ever seen mercury given in diarrhœa, I really think he cannot be serious! Surely Mr. K. need not be told that a disturbed state of the biliary secretion, is a very common exciting cause of diarrhœa. I conceive also that N. P. had no idea of exciting salivation when he recommended ' small alterative doses of mercury,' as Mr. K. conjectures; nor will mercury, administered with opium or rhubarb, or both, under such circumstances, augment the already too great excitement of the body. Quite the contrary, I conceive, will be its effects: it will allay irritation, by restoring the disordered secretion of the abdominal viscera to health." I shall observe, on the first part of this passage, that mercury is certainly not an appropriate remedy, nor have I ever seen it recommended in any of my medical researches, in such a case of diarrhœa, that has fallen under my observation. Where the irritation in the excremental canal did not proceed from a disturbed biliary secretion, but from the influence of cold obstructing the perspirable pores, and determining the flow of blood to the interior parts; and it had originated from the cause he specifies. I am of opinion, nion, that calomel alone, even in the smallest doses, would have a tendency to augment the already disordered action of the intestinal canal; but when combined with opium they have a separate effect. The stimulus caused by the hydrarg. submur. is counteracted and prevented from running off by stool, and facilitate its effects upon the morbid organ; but I cannot coincide with E. P. that pulv. rhei will have the same power. Opium and mercury joined may be highly judicious in such case as diarrhœa, where the cause is excited from a redundancy of bile, or any other, proceeding from an affection of the liver, but not in such form as N. P. observed in your former publication. Is there not a probability of the salivary glands being influenced by mercury, when exhibited in the manner N. P. recommended; I am induced to think there is, if not ejected by the bowels.

He again says, in his observations on a part of my letter, "I will answer part of it in the author's method; first, asking whether diarrhœa does not consist in a deranged state of the functions of the alimentary canal? and does the bile pass into the duodenum, for the purpose of assisting digestion? I thought, till now, that the stomach performed that office without the assistance of the bile." The author's remarks inclines me to think that he considers all diarrhœas as arising from that particular cause; I will beg leave to acquaint him, that his query requires but little explanation, as it is well known that this disease originates from a multiplicity of causes; as the influence of cold, passions of the mind, and several others too numerous to detail; therefore diarrhœa does not always consist in a deranged state of the stomach. In diarrhœa there is evidently a morbid increase of the peristaltic motion, which morbid increase is the effect of a variety of causes, applied either to the body in general, or acting solely on the parts affected: vide Dr. Thomas's Practice of Physic, p. 333, third edition. It is true that the bile has no communication with the stomach, unless regurgitated by a reverse peristaltic action of the intestines, which is of rare occurrence, and when it does it produces a disordered action in that organ. E. P. surely must be aware that the aliment undergoes some changes after passing into the bowels. In vindication of what I mentioned in my letter, the following is an extract from Dr. Thomas Maryat's Glossary to his Therapeutics, twentieth edition:—"The bile is a fluid which is secreted by the liver into the gall-bladder, and from thence passes into the intestines, in order to promote digestion."

I certainly should not have taken up my pen to answer any more anonymous observations, if my veracity had not been doubted. What objection can any gentleman have in disclosing his name, if he is acting upon such honorable grounds as will make him appear favorable in the public mind; until this is effected, I shall not think it worth my while to make any further vindication or remarks upon the subject. Whatever may be E. P.'s opinion respecting my knowledge of diseases, is very immaterial; but I will take the opportunity of informing him, that his remarks do not indicate him to be possessed of any extraordinary genius no more than myself. The attack made upon me, I consider weak and evasive; and so I have no doubt it will appear in the mind of every gentleman, who has the least liberality of sentiment. I am fully convinced, that the letters signed N. P. and E. P. were wrote, or at least instigated, by the same person; and, why that person should attack me in such an unfair and unhandsome manner, I am utterly at a loss to imagine.

I am, Gentlemen,

*Soham*, Your's respectfully,

* At the particular request of Mr. Knowles, we have inserted this letter *verbatim;* but we must now take leave of a controversy which promises so little interest to the public.—EDITORS.

