# Corrigenda

**Published:** 1812-06

**Authors:** 


### *CORRIGENDA.*

Page 374, line 20, for *of it*, read *of the cartilage*.

